# Dendritic Cell-T-Cell Circuitry in Health and Changes in Inflammatory Bowel Disease and Its Treatment

**DOI:** 10.1159/000442926

**Published:** 2016-03-16

**Authors:** Stella C. Knight

**Affiliations:** Imperial College London, Antigen Presentation Research Group, London, UK

**Keywords:** Tissue homing, Migration markers, Anti-inflammatory drugs, Vedolizumab, Dendritic cell plasticity, Blood markers for inflammatory disease, Cancer immunotherapy

## Abstract

**Background:**

Dendritic, antigen-presenting cells (DCs) determine not only whether lymphocytes produce different types of immune response but also tissue-homing profiles of lymphocytes they stimulate. For example, in health, mucosal DC stimulate T cells focused to home to the mucosa; DC/T-cell circuitry thus targets immune responses to specific tissue locations. Therapies being introduced for inflammatory bowel disease (IBD) include antibodies to gut-homing molecules such as α4β7 (Vedolizumab) used ostensibly to block gut-homing lymphocytes. However, such lymphocytes are dependent on the tissue specificity of DC that stimulated them.

**Key Messages:**

In health, blood DCs have the potential to home to multiple tissues including gut (α4β7+) and skin (CLA+). DCs have become gut-specific within the intestinal microenvironment stimulated partially by local retinoid to express α4β7 (mucosal homing marker) and/or CCR9 (ileal homing marker) in the absence of skin-specific indicators. They spread veiled extensions, sample their environment, acquire/process antigens, produce cytokines and initiate innate immunity. Myeloid DC also traffic to draining lymph nodes where compartmentalization of adaptive immune responses is determined by DCs from the site of antigen exposure which dictate the homing profiles of lymphocytes they stimulate. In IBD, site and activity of disease are reflected in changes in homing/activation of gut DCs and T-cells they stimulate and also, in greater gut specificity and activation of blood DC. Homing potential of DC can be modulated toward mucosa or skin by vitamins A and D, respectively. Infliximab or interleukin-6 can divert homing profiles toward skin, perhaps predisposing to skin involvement in IBD. Probiotic bacteria or their products can also change homing profiles of gut DC toward skin homing and away from gut.

**Conclusions:**

In conclusion, development of gut focused inflammation and its treatment relies on changes in DC tissue specificity; therefore, removal or diversion of gut-homing DC as well as T-cells is likely to be critical in prevention of gut-focused inflammation in IBD.

## Introduction

**Fig. 1:**
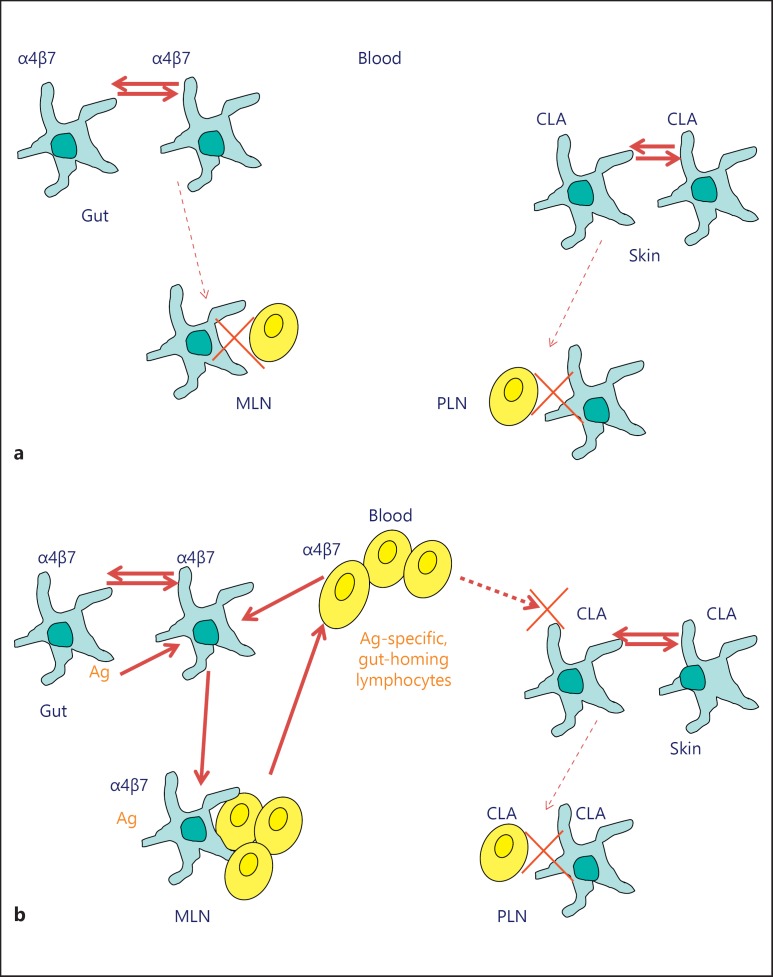
DC/T-cell circuitry. **a** In the resting state, most DCs within a tissue are tissue specific as illustrated from the expression of gut-specific α4β7 in gut mucosa or CLA in the skin. DCs will be exposed only to local tissue antigens and the migration of tissue-specific DCs to draining lymph nodes is minimal. Consequently, low numbers of lymphocytes are clustered and activated in the lymph node and there is little development of tissue-specific lymphocytes or of their release into the circulation. **b** Exposure of tissue to foreign antigen (Ag) in the gut is illustrated. Antigen is passed between DC and recognition of difference or potentially damaging effects promotes activation and migration of DC to draining lymph node. Antigen-specific gut DC within the draining lymph node will cluster and activate Ag-specific, gut-homing lymphocytes that are released into the circulation. These cells will home back to the gut toward the ligand for α4β7, mucosal vascular addressin cell-adhesion molecule 1, expressed by post-capillary endothelial cells in intestinal lamina propria. Specific targeted immunity to the foreign antigen in the gut will result. These cells will not have homing potential to other tissues such as the skin. Disease states, exposure to some bacteria or treatments for IBD can alter the activation and tissue-specificity of DC and subsequently of the lymphocytes they stimulate; such changes can change the activity or tissue restrictions of inflammatory disease or the immune responses to them.

Dendritic cells (DCs) determine the tissue specificity of the lymphocytes they stimulate [1]. Immune activity can, thus, be envisaged as a series of circuits of DCs and lymphocytes, each circuit with an established tissue ‘postal or zip code’ that determines their site of activity (fig. 1). However, this neat pattern may hold in health but falls down in the face of disease and its treatment.

An indication of acquired tissue specificity of human DC was first determined in early work on reactive arthritis following genital tract infection with *Chlamydia trachomatis*. Elementary bodies of *C. trachomatis* bind to collagen, an association believed to underlie the development of arthritis occurring in the collagen rich environment of the joint. There was a prevailing view that cross-reactive stimulation of immunity to collagen occurred because of its association with chlamydia and caused accumulation and/or stimulation of collagen-specific T-cells within the joint. However, DCs in the joints, but not systemically, carried the chlamydial antigens in post-chlamydial arthritis. By contrast, T-cells present in the joint merely reflected the systemic increase in chlamydia-specific T-cells that followed the genital tract infection [2]. In exact contradiction to the prevailing view, my interpretation was that there was non-responsiveness to a self-associated antigen on DC in the joint; there are precedents for non-responsiveness to foreign antigens linked to self-antigens [3,4]. This non-responsiveness meant that DCs bearing the chlamydial antigen persisted, continued to produce innate immune effects locally in the joint but did not migrate from the joint to lymph nodes to stimulate a local adaptive immune response. Together with the lack of activity of the circulating, chlamydia-specific T-cells to remove chlamydial antigen from the joint, these unexpected findings indicated the presence of a localized immune focus of non-responsiveness involving both DCs and T-cells. The observations spawned the concept of local circuits of immune responses involving both DCs and T-cells. The formal demonstration of such circuitry [1] has been followed by my aim of developing effective, tissue-specific, DC/lymphocyte circuits of responsiveness or of non-responsiveness/tolerance to manipulate responses to particular antigens for effective control of immunity. A circuit of non-responsiveness would preserve the integrity of a tissue expressing self-antigens or self-associated antigens whilst a responsive circuit would not only remove unwanted foreign antigenic incursions but also act as an immunological barrier to the growth of tissue at inappropriate sites (including tumors -unless they carried local self-antigens). These concepts are beginning to be understood and are relevant to the study of mucosal immunity and to understanding the development of IBD and its treatment.

The knowledge of specific T-cell homing markers and their ligands [5,6] was instrumental in being able to determine the involvement of DC in tissue homing of T-cells, and this knowledge allowed formal demonstration of the hypothesis that DCs were involved in determining tissue homing [1]. Thus, DCs from the gut or the gut-draining lymph nodes stimulated the development of gut-homing lymphocytes whilst the peripheral DC stimulated skin homing lymphocytes [1,7,8,9]; the source of the lymphocytes did not influence the migration potential [1]. Parallel studies showing the rapid acquisition of tissue-specific homing phenotypes by CD4 T-cells activated in cutaneous or mucosal lymphoid tissues supported the idea of local involvement of tissue-specific elements during the T-cell activation process [10].

Such concepts of responsiveness and non-responsiveness in different circuits of DCs and T-cells, and the involvement of innate and adaptive arms of immune response, may inform understanding of the processes underlying the role of antigens within the gut such as food and the gut microbiota that could either promote or discourage inflammation in the gut. Direct evidence of the critical importance of site of sensitization in relation to the site of inflammation induced by gut antigens in humans in IBD was obtained from early studies of sensitivity to food antigens in Crohn's disease. In Crohn's disease, there is increased permeability of the gut mucosa predisposing to the breaching of this barrier by food antigens as well as by the microbiota. The induction of systemic sensitivity to such antigens was, therefore, studied in patients and controls by examining the development of recall or memory T-cell responses to a selection of antigens that are met in the gut. Antigens studied were extracts of cow's milk, cereals, cabbage group, citrus fruits, peanut group, yeast and bacteria. In Crohn's disease but not controls, the reactivity of peripheral lymphocytes to these antigenic extracts, particularly multiple sensitivities, was common [11] (fig. 1b). Despite this evidence of systemic sensitization, inflammation in response to skin challenge was negative; however, inflammation on intra-rectal challenge with these antigens, often to multiple antigens, was again common in the Crohn's patients but not in controls [12]. The relationship between the site of sensitization and site of recall/inflammation was established indicating the relevance of compartmentalization of immune responses in gut inflammation in response to intestinal antigens in Crohn's disease. Thus, identification of sensitivity to particular gut antigens in IBD requires testing at the mucosal site or via gut-specific elements systemically.

## Homing Marker Expression on DC

The finding that the tissue source of the DC determined the migration pathways of lymphocytes they stimulate indicated that the DC themselves must have tissue specificity. The majority of blood T-cells, in health, express α4β7 in the absence of skin homing markers indicating that they have been ‘educated’ in the mucosa and have the capacity to home to the ligand mucosal vascular addressin cell-adhesion molecule 1 expressed by post-capillary endothelial cells in intestinal lamina propria. A smaller percentage of T-cells express skin homing markers without gut-homing potential. By contrast, around 80% of blood DCs in health have both gut and skin homing potential as indicated from expression of both α4β7 and CLA, respectively [13]. Since the major evidence from animal models is that DC do not recirculate but die in the lymph nodes [14,15], the multi-homing potential of blood DC is likely to be necessary to facilitate the distribution to different tissues of DC emerging from the bone marrow into blood. Once within the tissues, DC exposed to antigens locally will continue a pattern of maturation of chemokine receptors that helped to localize them to these different tissues and upregulate the chemokine receptor CCR7 (giving responsiveness to CCL19/21) which promotes homing via the afferent lymph to the lymph nodes [16]. Here, they can stimulate tissue-specific responses in naive lymphocytes that also locate there by virtue of their CCR7 expression.

Once the DCs reach the skin or gut, local environmental factors promote their tissue specificity; gut DC remain largely α4β7 positive but lose skin homing receptor for E-selection, CLA, although some colonic but not ileal DC may maintain or acquire some skin-homing potential [8,17]. Skin specificity without gut potential is found in many DCs in the skin [13]. A major instigator of the gut specificity for DC is retinoic acid which can also inhibit the T-cell stimulatory function of DC [18,19,20], albeit very low doses of retinoid may promote T-cell stimulation [18]. An effect of retinoic acid on monocyte-derived DC (MoDC) in vitro is to upregulate α4β7 and promote production of TGFβ and interleukin (IL)-6 [19]. However, MoDC differ from normal circulating blood DC in their tissue specificities. The blood monocytes themselves from which MoDC are derived, unlike DC, lack expression of both CLA and α4β7 but express CCR9, an ileal homing marker, and CCR5 which gives the capacity to migrate to inflamed tissues [20]. Following the culture period to obtain MoDC, the cells lack migration markers and could be said to be ‘homeless’; such lack of tissue direction may be a fundamental problem when trying to use MoDC therapeutically, perhaps underlying the disappointing results of attempted in vivo treatments with MoDC, particularly for tumors. The appropriate activation of immunity in vitro may not be realized in vivo if DCs are not equipped to direct immune responses to the required location. However, stable induction of α4β7 could be promoted in MoDC to provide the potential for migration to mucosal tissue [20]. Evidence that this process of inducing gut specificity occurs within the human gut environment in health was obtained by showing that blood DC exposed to the supernatants of normal human colonic biopsies upregulate both α4β7 and CCR9 on DCs, an effect which is prevented by blocking retinoic acid activity [13]. CCR9 is also a homing receptor for plasmacytoid DCs travelling to the ileum as well as for the myeloid DCs [21]. A proportion of myeloid DCs from the ileum, and to a smaller extent those from the distal but not the proximal colon, express CCR9 and stimulate the production of CCR9-positive T-cells licensing them to migrate to CCL25/TECK expressed on ileal epithelial cells [17,22]. Another migration marker involved in migration of both monocytes and DC to the gut is CCR2 [22]. In contrast to the functions of retinoic acid, vitamin D may influence leukocyte homing toward the skin.

## Changes in IBD

There is increasing information describing the subsets and properties of human intestinal DC [17,22,23,24,25]. Differences are now being defined in health between DC in ileum and colon [17] and between proximal and distal colon [22] which make the interpretation of data difficult where these locations have not been defined. In any case, detailed descriptions of alterations in human intestinal DC in IBD and analyses of homing marker expression on different subsets of intestinal DC in IBD are rare [23,26,27,28,29]. A predominant cytokine in the inflamed gut in ulcerative colitis is IL-6 which could promote skin homing in DC and T-cells they stimulate, an effect also reported with supernatants and cells of biopsies from inflamed areas of the gut [30,31]. Such an effect could possibly fuel skin involvement in some patients. However, despite this observation, in other studies, where colonic DC were collected as cells migrating from biopsies from ulcerative colitis patients, these DC induced lower expression of the skin homing markers CLA and CCR4 on stimulated T-cells compared with their healthy counterparts. These gut DC from ulcerative colitis patients also induced enhanced expression of the ileal homing marker CCR9 on stimulated allogeneic T-cells; expression of β7 integrin was initially high and remained so. Thus, the gut DCs in these ulcerative colitis patients induced a greater T-cell focus toward the gut and away from the skin suggesting promotion of increased gut-specific activity; the activity was accompanied by increased IL-4 production and a loss of IL-22 and IFNγ [28]. It is worth remembering, however, the great plasticity of DC so that single snapshots of gut DC activity will not provide a coherent picture and apparent effects on T-cells may be offset by the presence of persistent memory T-cells with a predetermined migratory potential.

A surprising finding is that blood DCs can act as a window on site and activity of disease in IBD; both the DCs and the T-cells in the blood in IBD can reflect the site of the disease within the gut and its activity. The DCs give a clearer picture of ongoing activity since expression of markers on activated DCs able to stimulate T-cells is not confounded by the persistence of homing markers such as α4β7 on pre-existing, long-lived, memory T-cells. The site of disease and its activity can be identified from changes in the migration markers and activation markers on blood DC. Thus, increases in CCR9 on blood DC concomitant with loss of skin homing potential is evident in many adult Crohn's patients and in a smaller proportion of pediatric Crohn's patients [32,33]; this blood DC change reflects the presence of ileal disease. There is also a loss in Crohn's patients of the double skin and gut homing DC population which is replaced by cells showing only gut specificity with high β7 expression - that is of DC resembling those normally only found in quantity in the gut [33]. The mechanisms producing this effective change in DC life history are unknown but include the possibility of recirculation of DC that have located to the gut, been stimulated, travelled to the lymph node and then recirculated via the efferent lymph; the gut-homing phenotype and the presence of CCR7 that favors lymph node homing on blood DC makes the recirculation idea likely. Alternatively, changes in systemic cytokine profiles that affect homing activity may occur.

In Crohn's disease with concomitant erythema nodosum, changes in the presence and acquisition of a skin homing profile in γδ T-cells was observed and resolved following successful treatment with steroids [34]. Thus, different subsets of T-cells may be differentially regulated in diseases reflecting different sites of disease, but the types of DC that may be involved in their stimulation has not always been determined. However, it is increasingly apparent that changes in the expression of homing markers and the activation profiles in blood DC and the lymphocytes they stimulate may contribute to the diagnosis and determining treatment options for IBD.

## Treatment-Induced Changes in DC Homing Potential

In some patients, effective therapy is achieved by blocking gut-homing T-cells using natalizumab (anti-α4) and particularly using vedolizumab (anti-α4β7); the latter avoids the potential dangers of interfering with brain tropic α4β1-positive cells, a possible risk with natalizumab [35]. Such successful approaches to therapy underline the importance of DC/T-cell circuitry in IBD.

However, other biological agents also alter the migration potential of DC and the lymphocytes they stimulate. The probiotic *Lactobacillus casei* was effective in changing the DC stimulatory potential of T-cells by promoting a homeostatic profile and making DC once again double positive for both skin and gut-homing markers; however, it failed to effect change in imprinting of CCR4 or of the gut-homing indicators CCR9 or β7 [31]. An exciting development is the discovery that some products of probiotic bacteria - or ‘postbiotics’ - may have therapeutic potential [36]. We have identified one such bacterial peptide, STp, from *Lactobacillus plantarum* which may have therapeutic value in IBD in part via its effects on homing profiles of DC and lymphocytes they stimulate. STp is a serine-threonine rich peptide which lacks cleavage sites and, therefore, resists breakdown by gut proteases. STp has anti-inflammatory effects on DC and reverses changes identified in gut DC studied ex vivo from ulcerative colitis patients. However, STp also changes the homing profiles of DC; through effects on the DC, STp instructs T-cells not only to reduce gut specificity but also increases their potential for migrating to the skin [26,37]. Such a diversionary tactic represents a novel way by which bacteria avoid detection and elimination by the adaptive immune system. T-cells stimulated by DC which have this changed tissue specificity will be sent on a wild goose chase to the skin where it is likely that they will not encounter the sensitizing bacteria since this site lacks intestinal microbiota. A by-product of diversion to the skin may, however, be the propensity for producing skin manifestations of disease where activated DCs or T-cells sent to the skin may initiate inflammation through local innate effects. Another treatment which diverts lymphocytes to the skin is Infliximab. T-cells increase their skin homing profile and changes may again determine the skin involvement that can follow infliximab treatment [38].

Enteral nutrition in pediatric Crohn's is an effective treatment for a high proportion of patients and, in parallel with the therapeutic effects, reverses many of the defects and changes in blood DC in patients with colonic but not ileal disease. The latter non-responsive group with ileal disease can be identified by high CCR9 on blood DC. The enteral nutrition, when successful, normalizes the tissue-homing profile of the blood DC so that they once again show little sign of activation and have both gut and skin homing potential commensurate with that seen in health.

## Conclusions

The evidence for DC/T-cell immune circuits is now overwhelming and indicates that the normal immune system can be neatly compartmentalized into tissue-specific cell circuits; such a circuit will provide tissue-specific response to local foreign antigens or lack of inflammatory response to self-antigens and, probably, to self-associated, antigens for that tissue. Evidence for changes in these circuits is increasingly found in IBD which results in greater focus of immune activity toward the gut. Furthermore, reflections of this change of focus and activity can often be identified systemically as well as in the gut itself; indeed, the DC in the blood can provide a window suggesting both the location within the gut of ongoing immune activity and the level of activation which are indicated by DC homing markers and maturation/activation status respectively. The picture may be reflected in the circulating memory lymphocytes but is more difficult to gauge due to long-term lymphocyte survival and memory of past history within lymphocyte populations. Despite the growing evidence for the importance of the DC/lymphocyte axis in the development and control of immune responses, including those in the gut, therapeutic control of immune activity is still predicated on diverting or removing effector lymphocytes systemically or from migration to the gut. Tackling more directly the instigators of the immune localization, the DC, may provide a further step forward for treatment. Despite the focus and importance of effector T-cells, it is becoming evident that effectiveness of many treatments used for IBD may stem from changes in the DC which reduce the focus of immune activity from the gut and, generally, promote DC homing potential toward the skin. Further detailed analysis of the underlying and multiple mechanisms controlling the DC/lymphocyte circuitry will lead to more efficient methods of controlling immune activity; if localized tissue-specific, immune manipulation can be achieved it should remove the need for blanket immunosuppressive therapies and facilitate the development of tissue-specific control of immunity in IBD. When we can upgrade our cell-based postal/zip code system from general area codes to satnav precision, individualized tissue-specific treatments should become achievable.

## Acknowledgement and Disclosure Statement

Stella Knight is a joint holder of a patent for the use of the bacterial peptide STp and is funded by the Biotechnology and Biological Sciences Research Council UK BB/J004529/1.
